# Professional socialization of hospital nurses: A scale development and validation study

**DOI:** 10.1186/s12912-022-01169-6

**Published:** 2023-01-04

**Authors:** Seongmi Moon, Soo Jung Chang

**Affiliations:** 1 Department of Nursing, College of Medicine, University of Ulsan, 93 Daehak-ro, Nam-gu, Ulsan, 44610 South Korea; 2 Department of Nursing, Gangneung-Wonju National University, 150, Namwon-ro, Heungeop-myeon, Wonju, 26403 South Korea

**Keywords:** Nurse, Professionalism, Psychometrics, Reproducibility of results, Validation study

## Abstract

**Background:**

Tools currently available to measure professional socialization are outdated or could not reflect various properties of professional socialization of nurses. Thus, the objective of this study was to develop and validate a professional socialization measurement instrument for hospital nurses.

**Methods:**

Fifty-two items were initially extracted from literature reviews and in-depth interviews with 32 nurses. After content validity testing, 48 items remained. They were used to survey 881 hospital nurses in Korea in the testing phase for construct validity and reliability.

Results: Four factors (21 items) were extracted: ethical practice and reflection, perception of respect and recognition, clinical competency based on leadership, and desires and motivation for professional development. These factors demonstrated good construct validity and reliability. Correlation coefficients of professional socialization with professional value, compassion satisfaction, secondary traumatic stress, and burden were 0.58 (*p* < 0.001), 0.70 (*p* < 0.001), − 0.08 (*p* = 0.014), and − 0.56 (*p* < 0.001), respectively. Reliability had a Cronbach’s alpha of 0.95. Test-retest reliability intraclass correlation coefficient was 0.90.

**Conclusions:**

The four professional socialization scale factors in this study reflected attributes of knowledge, skills, values, and professional roles. Professional socialization of nurses can be continuously developed by emphasizing elements of the professional socialization scale in nursing education programs. Nursing managers should help nurses go through the professional socialization process. The professional socialization scale will serve as a tool for developing careers of hospital nurses.

## Background

Nurses comprise the largest proportion of the healthcare workforce. Professional nurses ensure the quality of care and patient safety. They are essential elements of healthcare systems. For nurses to fulfill their roles in healthcare systems, they undergo professional socialization (PS), a process of acquainting oneself with their professional roles and gaining professional identity [1]. PS is the process by which nurses internalize their knowledge, attitudes, skills, values, and norms of the profession to develop a nursing professional identity [2]. It is an ongoing, lifelong process [3].

The importance of PS of nurses has been studied. Its outcomes include formation of professional identity and professional development [4]. Successful transition from a nursing student to a registered nurse depends on adequate socialization. Nurses who do not achieve PS in nursing education programs may face challenges, especially during early days of working [1]. In this context, PS can be an indicator that can consistently evaluate whether a nurse is ready or able to perform a professional role.

Since the level of PS differs depending on where the nurse is at the time and the nursing situation [2], evaluating PS presents a way to assess the outcome of nursing education in school or nursing practice in the field. Brown et al. [5] have identified the role of the clinical teacher in seven domains through a study investigating the role of a clinical teacher in student nurse PS. The PS instrument developed using these seven domains was applied to nursing students and clinical teachers at various universities, showing that the clinical teacher was an important adjunct socializing agent in the PS process of nursing students [6]. Maintaining a stable and quality nursing workforce is a significant public health goal. The importance of PS should not be underestimated in a context where nursing has high turnover and attrition rates [5]. Therefore, it is necessary to evaluate the PS of nurses and identify factors that hinder PS to raise the status of nursing profession in healthcare systems and ensure the quality of patient care.

The PS construct has attributes of knowledge, skills, attributes, values, norms, identity, and processes. However, when evaluating attributes, the operational definition was different in each study. Measurement methods were also inconsistent. Although previous studies on PS in nursing have evaluated some aspects of PS, assessment tools were outdated. In addition, some tools intended for other purposes were adapted to measure PS [6]. In Korea, studies on the PS of nursing students have been performed since 2000 using the PS scale developed by Du Toit [7] without any validation tests. The PS scale of Du Toit [7] is divided into two categories: value and personality. A recent study [8] that measured the PS of nursing students using the Du Toit [7] scale showed that the item structure was different from the original tool through validation and suggested the need to develop a PS measurement tool. Although there are a few studies on the PS of hospital nurses in Korea, there is no appropriate tool to measure PS. Furthermore, some tools focused on PS in nursing [6, 7] were developed for students, not nurses.

PS refers to taking actions within cultural norms [9]. It is meaningful to develop an empirical PS measurement tool based on existing research results known to reflect clinical experiences of hospital nurses within the Korean cultural norm. Thus, the objective of this study was to develop a PS scale (PSS) based on data extracted from clinical experiences of hospital nurses in consideration of the Korean environment and healthcare system. The PSS developed in this study is expected to help plan educational programs to improve the PS of clinical nurses and nursing students by identifying the level of PS. The PSS can identify socialization needs of professional nurses. It can be used to check the effectiveness of on-the-job training. In institutions, PSS can be used to develop strategies to recruit and retain nurses. A high professional socialization level among nurses will ultimately lead to improved quality of patient care.

## Methods

This was a methodologic study to develop and validate the PSS for registered clinical nurses according to the scale development process suggested by DeVellis [10]. This study also referred to COnsensus based Standards for the selection of health Measurement INstruments (COSMIN) [11]. Samples used at each stage were independent of each other.

### Item generation

We reviewed studies on PS concept analysis [2], the model [12], literature review studies [13, 14], and measurements [5–7] to understand the PS concept and its structure and to develop interview questions to construct a pool of items. Based on such review, we have conceptualized PS as follows. PS is a lifelong process of acculturation, the acquisition of knowledge, skills, professional values, and professional role, which develops into a sense of belonging and builds professional identity. In the process, learning and adaptation take place. Competence and proficiency are acquired.

Face-to-face or focus group interviews were conducted with questions asking about changes that had occurred in nurses’ knowledge, skills, attitudes, beliefs, and values during clinical practice. Thirty-two participants with various clinical experiences were selected for the interview. We directly contacted participants to form a focus group or selected individual participants using a snowball sampling. Of these 32 participants, 18 participated in focus group interviews and 14 participated in face-to-face interviews. Each focus group consisted of 3–5 nurses, with two groups (seven nurses total) having less than two years of clinical experience and three groups (11 nurses total) having 2–6 years of clinical experiences. The 14 nurses who participated in face-to face interviews had 2 to 20 years of clinical experience.

All interviews were recorded and then transcribed. While reading transcribed manuscripts, we extracted 159 items that were consistent with the concept of professional socialization. These items also appeared in literature reviews. After arranging overlapping content among these 159 items, 52 items were extracted. Responses were scored on a 6-point Likert scale (1 = strongly disagree, 6 = strongly agree). No intermediate responses were allowed to obtain more varied item averages while avoiding a centralization tendency.

### Content validity test

Nine nursing professionals who were educators in schools or hospitals, clinical nurses, and clinical administrators with Ph.D. degrees participated in content validity testing of the preliminary 52 items. A 4-point Likert scale was used to evaluate content validity. When evaluating content validity, nurses were asked for ways to improve the expression of unclear, irrelevant, or inappropriate items or to specify items that they did not believe were relevant. The criterion of Item Content Validity Index (I-CVI) was 0.78, indicating that the proportion answered “relevant” or “very relevant” was 78%. In the first test, the I-CVI of 52 items ranged from 0.56 to 1.00. The scale content validity index (S-CVI) was 0.86, indicating that the average I-CVI of the 52 items was 0.86. After deleting 4 items with I-CVI of 0.56–0.67 (under 0.78), a second content validity test with 48 items was performed by another three nursing professionals. Its S-CVI was 0.91. A small number of experts—about three to five—can evaluate the revised item set [15]. Thus, in the second round of this study, three experts evaluated the validity.

### Face validity test

A face validity test was performed by 11 nurses working at a general hospital. They were purposively sampled in consideration of their clinical experience: three nurses with less than 2 years of clinical experience, three nurses with 2–6 years of clinical experience, three nurses with 6–10 years of clinical experience, and two nurses with more than 10 years of clinical experience. We gave these 11 participants a PSS questionnaire and asked them to whether the meaning of the item was clear and understandable and whether there was any ambiguity or difficulty in responding. Results showed no problems in understanding or responding to the 48 items.

### Field test

#### Sample

Staff nurses, chief nurses, nursing unit managers, and team (unit grouping with similar characteristics) managers at general hospitals were included. Nurses with a higher position than managers were excluded. According to the recommended sample size for the factor analysis of 10 subjects per item [15], 480 subjects were needed for the 48 preliminary items. Since exploratory factor analysis (EFA) and confirmatory factor analysis (CFA) were planned, 960 subjects, twice as much as necessary, were needed. Considering a drop-out rate of 20%, a sample size of 1200 was planned.

#### Data collection

After obtaining a nationwide list of hospitals with over 300 beds from the Korean Hospital Nurses Association (KHNA), a number of subjects proportional to the number of nurses in each region was planned. We contacted nursing departments of hospitals on the list received from the KHNA, explained the study purpose and methods, and received permission for data collection. We sent questionnaires, informed consents, and participant recruitment notices to nursing departments by mail. Each nursing department posted a recruitment notice describing the purpose of this study, the criteria for selecting and excluding study subjects, and the method of participating in this study. Nurses wishing to participate in this study received and completed a consent form and questionnaire and sealed them in an addressed envelope. These data collection procedures were applied consistently at all sites.

A total of 895 nurses from 23 hospitals in 10 of 16 administrative regions in South Korea participated in this study. Of these subjects, 68.8% were from four metropolitan cities. Excluding questionnaires with missing values, a total of 881 questionnaires were analyzed. The sample consisted of 67.1% staff nurses, 17.4% chief nurses, and 15.5% nursing unit or team managers. Of the sample population, 97.0% were females. The mean age of all subjects was 34.50 years (standard deviation (SD): 8.96 years; range, 22–57 years). Their mean clinical experience was 11.02 years (SD: 9.09 years; range, 1 month – 37 years). Of them, 464 (51.7%) had experience with a preceptor. Among subjects, 480 were randomly assigned for EFA and 401 were assigned for CFA using a case random sampling method of the SPSS 24 program.

#### Construct validity and reliability test

SPSS 24 and Analysis of Moment Structure (AMOS) 24 were used to evaluate construct validity and reliability of the PSS. Descriptive statistics such as mean, SD, skewness, and kurtosis were calculated for basic item analysis. Pearson’s correlations were assessed to confirm the inter-item correlation and corrected item-scale correlation (items were removed in total scale score calculations). Criteria for item adoption were an inter-item correlation of 0.3–0.7 and 0.3 or higher for item-scale correlation [15].

To identify the structure of the PS concept, EFA was performed using maximum likelihood and oblique rotation. Before EFA, Kaiser-Meyer-Olkin (KMO) test and Bartlett’s test of sphericity were performed to determine data suitability for factor analysis. Factor extraction criteria were: an initial eigenvalue of 1.0 or higher, factor loading of 0.4 or higher, and total variance of 60% or higher [15]. Items with a difference of less than 0.2 in cross-factor loadings were deleted. Multi-trait scaling techniques were used for factors and items from the EFA to evaluate item internal consistency and item discriminant validity [16]. Item internal consistency was evaluated based on the correlation between an item and the factor to which the item belonged. The desired value was equal to or greater than 0.4. Item discriminant validity was satisfied when the correlation between an item and the factor to which the item belonged was two standard errors or higher than the correlation between the item and other factors.

CFA with maximum likelihood estimation was done to confirm the PSS structure by EFA. Fitness of the PSS structure was verified if the ratio of χ^2^ to the degrees of freedom was smaller than 3:1 [17], the Tucker-Lewis index (TLI) and comparative fit index (CFI) were close to 0.95, the standardized root mean squared residual (SRMR) was close to 0.08, and the root mean square error of approximation (RMSEA) was close to 0.06 [18]. Average variance extracted (AVE) and construct reliability (CR) were calculated for convergence validity. Convergent validity was fulfilled by an AVE of 0.5 or higher and a CR of 0.7 or higher [19]. We compared AVE values to squares of correlation coefficients between factors to verify discriminant validity of the PSS. AVEs should be larger than squares of correlation coefficients for discriminant validity [20]. Whether ‘*r* ± 2SE ≠ 1’ (r, correlation; SE, standard error of covariance) was satisfied was also determined. The heterotrait-monotrait (HTMT) ratio of correlations was also calculated to assess the discriminant validity. An HTMT criterion of 0.9 was needed for the large sample size in this study [21].

For hypothesis testing, the relationship between PSS and the Korean version of the Nurses Professional Value Scale-Revised (NPVS-R) [22] and the relationship between PSS and the Professional Quality of Life (ProQOL) scale [23] Korean version were analyzed. The Korean version of the NPVS-R is a 5-point Likert scale that consists of 26 items including five factors: human dignity, professionalism, innovation, contribution, and advocacy. In this study, Cronbach’s alpha of the Korean version of the NPVS-R was 0.93. The ProQOL scale Korean version is a 5-point Likert scale that consists of 30 items. It has three subscales: compassion satisfaction, secondary traumatic stress, and burden. Their Cronbach’s alphas in this study were 0.91, 0.76, and 0.83, respectively. Associations of PSS with job position, preceptor experience, and length of employment were also analyzed.

Cronbach’s alpha was calculated to evaluate the internal consistency reliability of PSS. For test-retest reliability, considering that retest intervals are generally recommended as 2–4 weeks because of memory, desire for consistency and rehearsal effects at short retest interval, and response shift at long intervals [24, 25], the intraclass correlation coefficient (ICC) was calculated with data surveyed twice at an interval of three weeks for 60 subjects in this study.

## Results

### Construct validity

#### EFA

Item-scale correlations of all 48 items were higher than 0.3. For inter-item correlations, item 20 showed correlation coefficients lower than 0.3 with 9 items. Thus, item 20 was deleted. We also deleted items 1, 7, 10, 23, 25, 40, 43, 46, 47, and 48 after reviewing redundancy between contents of items that showed inter-item correlation coefficients higher than 0.7. Mean scores of a total of 37 items used for EFA ranged from 3.83 (SD, 1.05) to 4.85 (SD, 0.78). The standardized skewness for 4 items (item 2, 17, 28, and 38) and the standardized kurtosis for 7 items (item 6, 9, 22, 28, 31, 35, and 38) were higher than 1.96 at a significance level of 5%.

In the first EFA, the KMO measure of sample adequacy was 0.97, which was exemplary for factor analysis [26]. Bartlett’s test of sphericity revealed a χ^2^ value of 13,132.74 (*p* < 0.001), which was also suitable for factor analysis. Communality values for the 37 items ranged from 0.41 to 0.78. Five factors explaining 65.30% of the total variance were extracted. EFA was conducted by deleting items that were not loaded by any factor, or loaded at two or more factors simultaneously, or items with a difference of less than 0.2 in cross-factor loadings. After performing the seventh EFA, 4 factors with 23 items meeting all conditions were extracted.

In multi-trait scaling techniques, item internal consistency was guaranteed as correlations between items an factors to which items belonged were higher than 0.4. Except for two items (items 16 and 24 from factor 1) out of 23 items, the value obtained by subtracting twice the standard error from the correlation with the factor to which the item belonged was higher than the correlation with other factors, verifying discriminant validity for 21 items. EFA was performed again because items 16 and 24 were less similar in content compared to other items in factor 1. In the final EFA, the KMO was 0.95. Bartlett’s test of sphericity revealed a χ^2^ value of 6306.37 (*p* < 0.001). Four factors with 21 items explaining 67.12% of the total variance were extracted (Table 1).

**Table 1:** Pattern Matrix of Exploratory Factor Analysis (Final Stage) (*N* = 480)

Factor and Item		Factor Loading			
		1	2	3	4
Factor 1: Ethical practice and reflection (EP)					
45	I take action to create a safe environment.	0.70	0.01	0.13	0.04
37	I share new knowledge and skills with fellow nurses.	0.70	0.00	0.06	0.03
42	I look back and reflect on the nursing I have performed.	0.69	0.01	0.12	0.06
29	I support my fellow nurses.	0.69	0.05	0.02	0.01
39	I empathize with the nursing clients.	0.68	0.08	0.04	0.01
21	I respect nursing clients as human beings with dignity.	0.66	0.07	0.01	0.11
36	I take ethical attitudes and behaviors as a professional nurse.	0.65	0.01	0.08	0.08
22	I make inferences and judgments based on evidence.	0.59	0.04	0.19	0.05
Factor 2: Perception of respect and recognition (RR)					
18	I know that professional nurses are trusted in our society.	0.03	0.92	0.08	0.02
19	I know that professional nurses are recognized in our society.	0.16	0.73	0.13	0.02
17	I am respected by nursing clients as a professional nurse.	0.04	0.60	0.21	0.12
Factor 3: Clinical competency based on leadership (CL)					
13	I show appropriate leadership in my position.	0.01	0.07	0.84	0.03
12	I am sensitive to the needs of nursing clients.	0.24	0.03	0.56	0.03
15	I actively participate in activities that change and improve the work environment and organizational culture.	0.08	0.13	0.52	0.18
8	I take the initiative in planning and performing nursing care.	0.17	0.02	0.48	0.20
11	I know and comply with the standards of the nursing profession.	0.24	0.00	0.44	0.15
Factor 4: Desires and Motivation for Professional Development (DM)					
4	I have a continuing need to learn as a professional nurse.	0.05	0.07	0.08	0.87
5	I am motivated to provide nursing care.	0.08	0.10	0.04	0.82
3	I apply new knowledge and skills to my nursing performance.	0.01	0.02	0.09	0.67
6	I perform nursing with a sense of duty.	0.19	0.06	0.05	0.60
2	I take pride in being a professional nurse.	0.02	0.19	0.08	0.57
Initial eigenvalues		10.51	1.45	1.13	1.01
Initial cumulative % of variance		50.03	57.00	62.32	67.12
KMO = 0.95; Bartlett’s test of sphericity: χ^2^ = 6306.37, df = 219, *p* < 0.001					

KMO = Kaiser-Meyer-Olkin

Factor 1 consisted of eight items, including ethical attitude, reflection, evidence-based practice, and respect for nursing clients and colleagues. They were named “ethical practice and reflection (EP).” Factor 2 consisted of three items on being recognized and respected as a professional nurse. They were named “perception of respect and recognition (RR).” Factor 3 consisted of five items on sensitive awareness of clients’ needs, proactive work performance, and leadership. They were named “clinical competency based on leadership (CL).” Factor 4 consisted of five items on a desire for learning and motivation for nursing. They were named “desires and motivation for professional development (DM).”

#### CFA

CFA confirmed the four-factor PSS constructed with 21 items (Table 2). Indices of fitness included a ratio of χ^2^ to the degrees of freedom of 2.52, a TLI of 0.94, a CFI of 0.95, and an SRMR of 0.04. RMSEA was 0.06, indicating reasonable fitness. AVEs ranged from 0.65 to 0.70 and CRs ranged from 0.85 to 0.94, indicating satisfactory convergent validity of the PSS. In terms of discriminant validity, squares of the correlation coefficients between factor 1 and factor 3 (0.78), between factor 1 and factor 4 (0.71), and between factor 3 and factor 4 (0.81) were higher than the highest AVE value. The *r* ± 2SE value was 0.82–0.96 between factor 1 and factor 3, 0.78–0.91 between factor 1 and factor 4, and 0.82–0.98 between factor 3 and factor 4. HTMT ratios of correlations ranged from 0.63 to 0.90. It was 0.9 between factor 3 and factor 4, which was a borderline value.

**Table 2:** Confirmatory Factor Analysis and Item Convergent-Discriminant Validity (*N* = 401)

Factor	Item	Estimate	Std. estimate	SE	AVE	CR	*r*^2^, HTMT				*r* ± 2SE			
							EP	RR	CL	NM		*r*	SE	±2SE
EP	21	1.00	0.68		0.65	0.94					EP-RR	0.60	0.03	0.54–0.67
	22	0.94	0.71	0.07							EP-CL	0.89	0.04	0.82–0.96
	29	0.89	0.65	0.07							EP-DM	0.85	0.03	0.78–0.91
	36	1.16	0.78	0.08										
	37	1.03	0.70	0.08										
	39	1.03	0.78	0.07										
	42	0.93	0.67	0.08										
	45	1.11	0.80	0.08										
RR	17	1.00	0.81		0.66	0.85	0.36, 0.63				RR-CL	0.66	0.04	0.59–0.74
	18	1.19	0.86	0.07							RR-DM	0.64	0.08	0.56–0.71
	19	0.97	0.73	0.06										
CL	8	1.00	0.79		0.68	0.92	0.78, 0.89	0.44, 0.68			CL-DM	0.90	0.04	0.82–0.98
	11	0.84	0.73	0.05										
	12	0.85	0.76	0.05										
	13	0.95	0.82	0.05										
	15	0.99	0.78	0.06										
DM	2	1.00	0.79		0.70	0.92	0.71, 0.85	0.41, 0.66	0.81, 0.90					
	3	0.83	0.77	0.05										
	4	0.94	0.79	0.05										
	5	1.04	0.84	0.06										
	6	0.99	0.76	0.06										
χ^2^ = 460.33, χ^2^/df = 2.52, TLI = 0.94, CFI = 0.95, SRMR = 0.04, RMSEA (90% CI) = 0.06 (0.055–0.069)														

AVE = average variance extracted; CFI = comparative fit index; CL = clinical competency based on leadership; CR = construct reliability; DM = desire and motivation for professional development; EP = ethical practice and reflection; HTMT = heterotrait-monotrait; *r* = correlation coefficient; RMSEA = root mean square error of approximation; RR = experience of respect and recognition; SE = standard error; SRMR = standardized root mean residual; Std. = standardized; TLI = Tucker-Lewis index

#### Hypothesis testing

Correlations of PSS with the Korean version of the NPVS-R, compassion satisfaction, secondary traumatic stress, and burden which were three components of the ProQOL scale Korean version were 0.58 (*p* < 0.001), 0.70 (*p* < 0.001), − 0.08 (*p* = 0.014), and − 0.56 (*p* < 0.001), respectively. PS measured by the PSS was positively correlated with the length of employment. There was a significant difference in PS according to job position and preceptor experience, showing higher scores for those with higher positions and preceptor experience. These results verified the construct validity through the hypothesis test (Table 3).

**Table 3:** Hypothesis Testing (*N* = 881)

	r / t / F (p) or Mean (SD)				
Variables	EP	RR	CL	DM	PSS
NPVS-R	0.59 (< 0.001)	0.37 (< 0.001)	0.53 (< 0.001)	0.55 (< 0.001)	0.58 (< 0.001)
Compassion satisfaction	0.62 (< 0.001)	0.55 (< 0.001)	0.59 (< 0.001)	0.66 (< 0.001)	0.70 (< 0.001)
Secondary traumatic stress	−0.11 (0.002)	−0.01 (0.736)	−0.10 (0.005)	−0.09 (0.008)	−0.08 (0.014)
Burden	−0.48 (< 0.001)	−0.45 (< 0.001)	−0.48 (< 0.001)	−0.53 (< 0.001)	−0.56 (< 0.001)
Length of employment	0.41 (< 0.001)	0.32 (< 0.001)	0.51 (< 0.001)	0.40 (< 0.001)	0.47 (< 0.001)
Job position					
Staff^a^	4.46 (0.56)	3.81 (0.86)	4.16 (0.64)	4.32 (0.67)	4.19 (0.56)
Chief^b^	4.68 (0.53)	4.04 (0.88)	4.61 (0.60)	4.67 (0.64)	4.50 (0.56)
Nursing unit manager^c^	5.08 (0.51)	4.56 (0.72)	5.03 (0.55)	5.01 (0.61)	4.92 (0.50)
Team manager^d^	5.29 (0.48)	4.92 (0.56)	5.29 (0.49)	5.20 (0.61)	5.18 (0.43)
	53.15 (< 0.001) a < b < c,d	34.19 (< 0.001) a < b < c,d	86.85 (< 0.001) a < b < c,d	48.31 (< 0.001) a < b < c,d	75.01 (< 0.001) a < b < c,d
Gender					
Female	4.60 (0.59)	3.98 (0.88)	4.38 (0.71)	4.49 (0.71)	4.36 (0.62)
Male	4.41 (0.65)	3.68 (0.98)	4.16 (0.67)	4.38 (0.78)	4.16 (0.58)
	1.55 (0.122)	1.61 (0.105)	1.52 (0.128)	0.75 (0.453)	1.60 (0.110)
Experience of preceptor					
Yes	4.64 (0.59)	3.97 (0.89)	4.49 (0.68)	4.53 (0.68)	4.40 (0.61)
No	4.54 (0.59)	3.95 (0.88)	4.23 (0.71)	4.43 (0.73)	4.29 (0.62)
	2.58 (0.010)	0.36 (0.722)	5.29 (< 0.001)	2.01 (0.044)	2.82 (0.005)

SD = standard deviation; EP = ethical practice and reflection; RR = experience of respect and recognition; CL = clinical competency based on leadership; DM = desire and motivation for professional development; NPVS-R = Nurses Professional Value Scale-Revised (Korean version)

#### Reliability

Cronbach’s alpha of the PSS was 0.95 (range, 0.84 to 0.90 for each factor). The ICC of the PSS was 0.90 (range, 0.77 to 0.79 for each factor) (Table 4).

**Table 4:** Test-Retest Reliability and Internal Consistency Reliability (N = 881)

Factor	ICC (95% CI) (*n* = 60)	Cronbach’s alpha (n = 881)
Ethical practice and reflection	0.79 (0.65–0.87)	0.90
Experience of respect and recognition	0.77 (0.62–0.86)	0.84
Clinical competency based on leadership	0.78 (0.64–0.87)	0.88
Desire and motivation for professional development	0.81 (0.69–0.89)	0.89
Professional socialization scale (total)	0.90 (0.84–0.94)	0.95

ICC = intraclass correlation coefficient

## Discussion

This study was conducted to develop and validate a PSS self-rating scale for clinical nurses. The PSS differs from the existing scale for nursing students [7] in that it includes not only one’s perspective on the value and norm of PS, but also collaboration with colleagues and daily work performance in real clinical situations.

The PSS consisted of 21 items and 4 factors. Factor 1 (EP) included 8 items regarding ethical attitude and reflection, evidence-based practice, respect for nursing clients, as well as collaboration with colleagues. It showed the highest explanatory power scores (50.03%). Ethical practice and reflection items were related to daily direct nursing practice by the clinical nurse, which contributes to the quality of practice settings, thereby positively affecting patient health outcomes [27]. These seemed to be reasons for the high explanatory power. Ethical behavior is the hallmark of a profession and a code of ethics that provides professional standards and guides nurses’ decision-making as they navigate ethical dilemmas frequently faced in clinical practice settings [27, 28]. Because reflection is fundamental for the socialization process and PS relies on the degree of self-reflection ability [4], reflection items can also be seen as appropriate questions to measure professional socialization. Items such as “sharing new knowledge and skills with fellow nurses” and “supporting my fellow nurses” show a professional competency regarding interacting with others and the teamwork necessary to solve problems, indicating that PS makes the person interact with the working environment and participate in interpersonal communication [14] or conversely, personal characteristics such as being open to collaboration as a team, sharing information, and readiness to work with peers toward common goals can facilitate socialization [1, 4].

Factor 2 (RR) included three items related to social evaluation of an individual. It showed an explanatory power of 6.97%. A professional has a clear identity and role in society and an associated recognition and endorsement by society for that unique role [29]. Being respected and recognized by clients and the public is essential for individual reward and motivation, which ultimately affects nursing performance improvement [30]. Thus, disrespectful relationships and lack of recognition force nurses to leave their jobs, whereas positive job performance feedback from patients and their families gives nurses a sense of calling and professional pride and motivates them to retain their jobs [31]. In a qualitative study on hospital nurse turnover in Korea [32], the importance of public/social perception was emphasized by participants because recognition of fair nurse compensation/treatment fostered pride and a positive job identity. Similarly, social respect and recognition of nurses can help them build identities as nurse professionals through PS [30, 33]. Therefore, perception of respect and recognition items can be regarded as important elements constituting PS.

Factor 3 (CL) included five items. It showed an explanatory power of 5.32%. These items such as proactive work performance, being sensitive to the needs of nursing clients, and active participation were similar to characteristics of leadership [34] and categories of innovation and visionary [27], which are foundations for nursing professional practice [28]. Leadership includes innovation and creativity. It shows people-oriented characteristics [34]. Professional nurses who challenge prevailing values and assumptions and reinforce that enhanced nursing practice can have a tremendous impact on patient outcomes contribute to changing traditional practices [27]. Clinical nurse leaders are crucial to the success of patient care initiatives as good leaders help produce good care [34]. For this reason, clinical competency based on leadership items well-reflected conceptual elements of professional socialization.

Factor 4 (DM) included five items closely related to the continuous desire for learning and motivation for nursing. It showed an explanatory power of 4.80%. These items were highly consistent with PS attributes such as the ongoing process of lifelong learning for professional growth and development and the process of learning new roles and adapting to them [2, 27]. Furthermore, enthusiasm and motivation are needed in the lifelong learning process. Salisu and colleagues [1] have found that internal motivation is critical in role acceptance and an essential element in succeeding despite challenges. In addition, eagerness and enthusiasm have been identified as contributors to successful socialization [1].

Meanwhile, social factors that influence the development of PS are gender, the image of nursing, and technology [28]. Items that reflected nursing as a calling, nurturing and feminine, service, and housekeeping remained dominant as Nightingale’s values on the Du Toit [7] scale were not included in the PSS. Black [28] has stated that nursing profession and education have been affected by gender-specific stereotypes for 160 years since Florence Nightingale worked to establish nursing as a career worthy of respectable women. However, in Korea, male students among total students who passed the National Nurses Examination accounted for 14.7% in 2020 and the cumulative number of male nurses exceeded 20,000 in the same year [35]. Furthermore, although nursing was recognized as a calling and the notion of service to humanity through nursing in the 1980s – 1990s [7, 36], the current appeal of nursing seems to provide economic and job security [28].

In the PSS, “applying new knowledge and skills” also differed from the previous scale [7]. As healthcare technology has advanced, its importance is growing in the nursing field because nurses can care for patients and make clinical judgments using various technical equipment and new technologies based on Internet systems [28]. Nurses as professionals should play an important role in developing and applying new knowledge and skills to improve nursing care and patient outcomes [28].

The strength of the PSS was that its construct validity and reliability were verified in a large sample. The significantly high correlation between PSS and the NPVS-R proved that PS was a process that could achieve a professional role with values and norms [1, 37]. Moreover, the positive correlation between PSS and compassion satisfaction and negative correlations of PSS with secondary traumatic stress and burden confirmed the relevance and differences between concept definitions in previous literature [23]. Significant differences in PS levels according to job position, preceptor experience, and length of employment meant that PSS could measure group differences by reflecting the conceptual characteristic that the PSS was a role-taking and ongoing developmental process [4]. The above elements were also factors significantly related to PS in a previous study [30]. Another strength was that the PSS had high internal consistency and excellent test-retest stability. As PSS showed significant correlations with NPVS-R and ProQOL in this study, PSS will have significant relationships with nurses’ professional values, professionalism, satisfaction, quality of life, stress, and burden.

### Limitations

In the PSS, the correlation between factors was high, and the discrimination between factor 3 (CL) and factor 4 (DM) was relatively unclear. This is a limitation of EFA and CFA, which are traditional construct measurement methods, and it is necessary to apply exploratory structural equation modeling (ESEM), an overarching integration of the best aspect of traditional EFA and CFA [38] to address these shortcomings. The lack of invariance verification as an internal structure assessment was also a limitation of this study. Since most of the nurses participating in this study were women, whether the PSS could be conceptually similarly interpreted in men was not confirmed.

In the data analysis process, responses with missing values were excluded, but potential careless responses were not detected. The PSS is not free from biases involving cognition, memory, and social desirability because it is a self-reported questionnaire.

The PSS was developed for hospital nurses and did not reflect the PS attributes of nurses working at sites other than hospitals. Therefore, the PSS needs to be revised and improved for nurses in other settings. The content validity test, including the face validity test, only included a small sample size. In this process, it is better if evaluators in the content validity test stage following item generation conduct a test with an appropriate number of samples (a minimum sample of 30) and include not only nursing professionals but experts from other related disciplines. Since the PSS was developed in a Korean sociocultural context, it needs to be verified in other cultures and modified accordingly.

## Conclusions

The PSS scale developed in this study consisted of 21 items and 4 factors. These 4 factors were ethical practice and reflection, perception of respect and recognition, clinical competency based on leadership, and desires and motivation for professional development. These factors reflected PS attributes of knowledge, skills, values, and professional roles. Moreover, the factor “perception of respect and recognition” was a unique result of this study. The PSS showed a significant change in PS according to professional aspects such as job position and experience with a preceptor. PSS is expected to serve as an indicator for developing careers of professional nurses.

## Data Availability

The datasets generated and/or analyzed during the current study are not publicly available due the informed consent did not include the provision of research data from participants to a third party, but are available from the corresponding author upon reasonable request.

## Abbreviation

AMOS: Analysis of Moment Structure
AVE: average variance extracted
CFA: confirmatory factor analysis
CFI: comparative fit index
CL: clinical competency based on leadership
CR: construct reliability
DM: desires and motivation for professional development
EFA: exploratory factor analysis
EP: ethical practice and reflection
ESEM: exploratory structural equation modeling
HTMT: heterotrait-monotrait
ICC: intraclass correlation coefficient
I-CVI: Item Content Validity Index
KHNA: Korean Hospital Nurses Association
KMO: Kaiser-Meyer-Olkin
RMSEA: root mean square error of approximation
NPVS-R: Nurses Professional Value Scale-Revised (Korean version)
ProQOL: Professional Quality of Life
RNAO: Registered Nurses’ Association of Ontario
RR: perception of respect and recognition
PS: professional socialization
PSS: professional socialization
S-CVI: scale content validity index
SD: standard deviation
SE: standard error
SRMR: standardized root mean squared residual
TLI: Tucker-Lewis index.
